# Risk stratification based on components of the complete blood count in patients with acute coronary syndrome: A classification and regression tree analysis

**DOI:** 10.1038/s41598-018-21139-w

**Published:** 2018-02-12

**Authors:** Xiaowei Niu, Guoyong Liu, Lichao Huo, Jingjing Zhang, Ming Bai, Yu Peng, Zheng Zhang

**Affiliations:** 1 The First School of Clinical Medicine, Lanzhou University, Lanzhou, Gansu China; 2 Department of Cardiology, the First Hospital of Lanzhou University, Lanzhou, Gansu China; 3 Baiyin Second People’s Hospital, Baiyin, Gansu China; 4 Gansu Key Laboratory of Cardiovascular Disease, Lanzhou, Gansu China

## Abstract

To develop a risk stratification model based on complete blood count (CBC) components in patients with acute coronary syndrome (ACS) using a classification and regression tree (CART) method. CBC variables and the Global Registry of Acute Coronary Events (GRACE) scores were determined in 2,693 patients with ACS. The CART analysis was performed to classify patients into different homogeneous risk groups and to determine predictors for major adverse cardiovascular events (MACEs) at 1-year follow-up. The CART algorithm identified the white blood cell count, hemoglobin, and mean platelet volume levels as the best combination to predict MACE risk. Patients were stratified into three categories with MACE rates ranging from 3.0% to 29.8%. Kaplan-Meier analysis demonstrated MACE risk increased with the ascending order of the CART risk categories. Multivariate Cox regression analysis showed that the CART risk categories independently predicted MACE risk. The predictive accuracy of the CART risk categories was tested by measuring discrimination and graphically assessing the calibration. Furthermore, the combined use of the CART risk categories and GRACE scores yielded a more accurate predictive value for MACEs. Patients with ACS can be readily stratified into distinct prognostic categories using the CART risk stratification tool on the basis of CBC components.

## Introduction

Acute coronary syndrome (ACS) includes a heterogeneous spectrum of diseases in terms of pathophysiological mechanisms, clinical presentation, and prognosis^1,2^. Risk stratification plays a crucial role in the management of patients with ACS^3^. Patients estimated to be at a higher risk may be managed with earlier and more aggressive treatment, whereas those with lower risk may be managed with less intensive treatment. The use of validated risk scoring methods, such as the Global Registry of Acute Coronary Events (GRACE) risk score^4^, has been recommended by the guidelines^3^. However, since the GRACE score reflects only some of the mechanisms related to outcome in ACS, biomarkers addressing other pathophysiological aspects of ACS could provide additional information. Recent studies reported that combining hematological indices with the GRACE score facilitated better prediction of future cardiovascular events in patients with ACS as compared to the use of the GRACE score alone^5–7^.

Although complete blood count (CBC) is a low-cost laboratory test that is almost universally used, it is often underused in regard to its risk predictive information. Several CBC components have been reported to independently predict adverse outcomes in patients with ACS, such as white blood cell (WBC) count^8,9^, neutrophil count^7^, hemoglobin^6^, red blood cell distribution width (RDW)^5^, and mean platelet volume (MPV) levels^10^. Although each individual CBC component might provide modest predictive ability, a risk stratification model, derived from combining variables in the CBC, could have synergistic advantages. A CBC risk score has been developed in patients undergoing coronary angiography for all-cause mortality^11^ and clinical morbidity endpoints^12^, and subsequently rederived in individuals with no cardiovascular disease history^13^. The CBC score, based on beta coefficients from a logistic regression model, was a powerful predictor of poor outcomes in patients with suspected cardiovascular disease, suggesting that combined use of CBC components can provide valuable additional risk information to clinicians. However, few studies have produced a clinically practical way of integrating various CBC variables to stratify risk in patients with ACS.

Classification and regression tree (CART) analysis is an innovative and powerful statistical technique where the most important predictors of outcomes are identified and patients are divided into different homogeneous risk groups^14^. CART analysis has been shown to allow reliable risk stratification for in-hospital mortality in patients with acute heart failure^15^ and myocardial infarction (MI)^16^. Moreover, compared with logistic or Cox regression models requiring a nomogram reference to calculate risk, the CART analysis produces a decision tree that is simple to interpret and apply at the bedside^14^. In the present study, CART analysis was performed to identify key CBC components and develop a risk stratification model. The incremental prognostic value of combining the CART risk model with the GRACE score was also determined.

## Results

### CART for CBC to stratify risk

**Figure 1:**
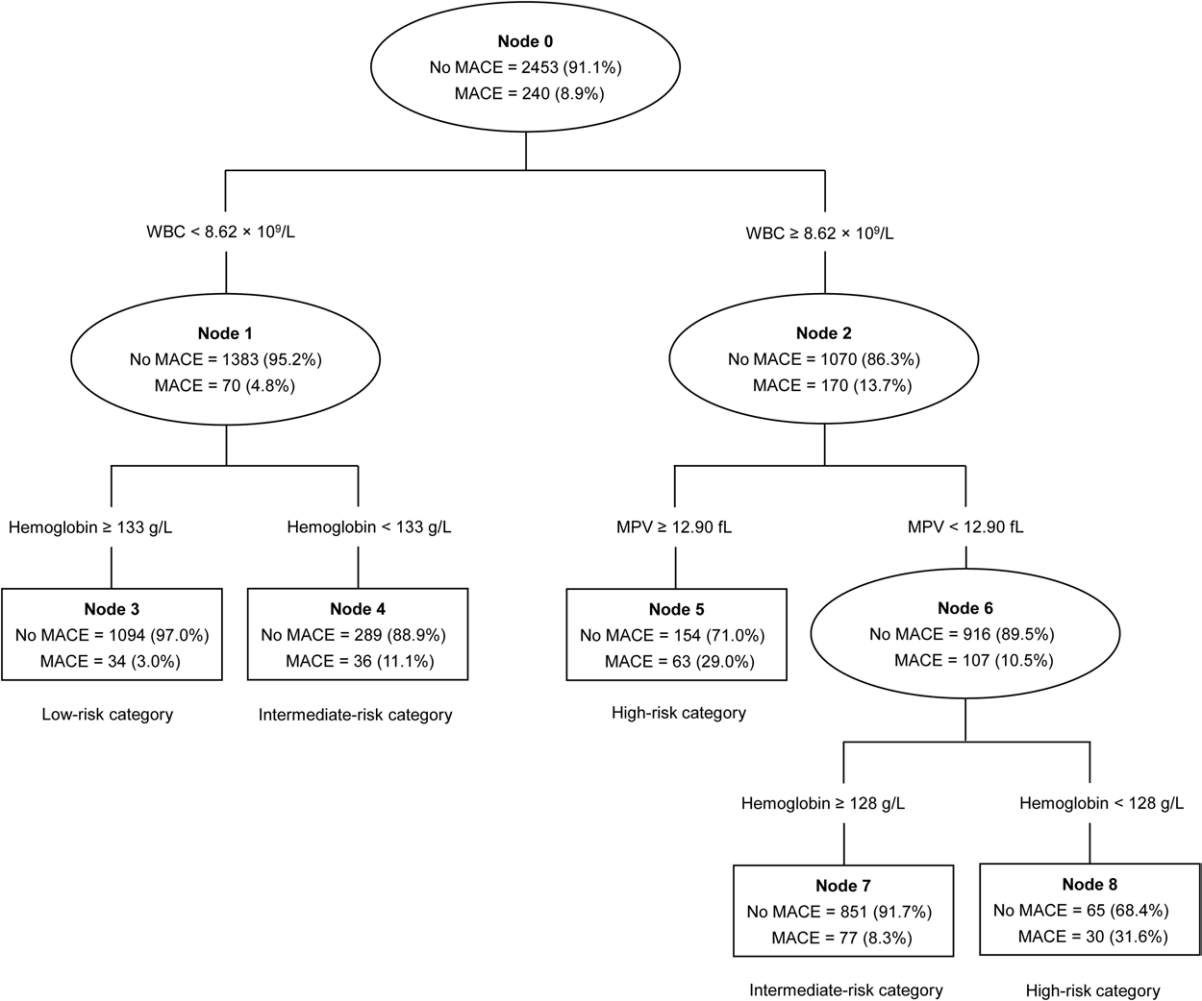
Predictors of 1-year major adverse cardiac events using the classification and regression tree model, and risk stratification for patients with acute coronary syndrome. Each predictor was written within line, and each node is based on the data available for each of the predictive variables presented. Each approval rate for each predictor is marked within ovals (intermediate node) or squares (terminal node). WBC, white blood cell; MPV, mean platelet volume; MACEs, major adverse cardiovascular events.

Of the 2,693 patients with ACS, 240 (8.9%, 95% CI 7.8-10.0%) had experienced major adverse cardiovascular events (MACEs) at 1-year follow-up. The CART method identified the WBC count from the 18 CBC metrics as the best single discriminator between those with and without MACEs. For the node with patients having a WBC count of less than 8.62 × 10^9^/L, a hemoglobin level of less than 133 g/L provided additional prognostic value. For the node with patients having a WBC count of 8.62 × 10^9^/L or higher (≥8.62 × 10^9^/L), the next best predictor of a MACE was MPV. For the node with patients having the WBC count of 8.62 × 10^9^/L or higher (≥8.62 × 10^9^/L) and a MPV level of less than 12.90 fL, a hemoglobin level of less than 128 g/L provided increased prognostic information. Based on the number of positive biomarkers in the CART analysis, patients were stratified into three risk groups: 1) low-risk with 0 positive biomarker (WBC count <8.62 × 10^9^/L and hemoglobin level ≥133 g/L), 2) intermediate-risk with 1 positive biomarker (WBC count <8.62 × 10^9^/L and hemoglobin level <133 g/L; or WBC count ≥8.62 × 10^9^/L, MPV level <12.90 fL, and hemoglobin level ≥128 g/L), and 3) high-risk with 2 positive biomarkers (WBC count ≥8.62 × 10^9^/L and MPV level ≥12.90 fL; or WBC count ≥8.62 × 10^9^/L, MPV level <12.90 fL, and hemoglobin level <128 g/L). Figure 1 depicts the final tree generated by the CART analysis and each child node of this tree.

### Baseline characteristics in the stratified patients with ACS

Baseline demographics and clinical characteristics of patients in the three risk groups are shown in Table 1. High-risk and intermediate-risk patients were more likely to be older, female and have a history of diabetes mellitus, advanced Killip class, cardiac arrest, elevated cardiac enzymes, ischemic ST-segment changes on electrocardiograms (ECG), and a diagnosis of ST-segment elevation MI (STEMI) when compared with low-risk patients. High-risk and intermediate-risk patients also had a higher GRACE score and worse clinical profile in terms of heart rate, systolic blood pressure, low density lipoprotein cholesterol, glucose, creatinine, and left ventricular ejection fraction. Additionally, there was a weak correlation between the CART risk categories and the GRACE score (r = 0.289, P < 0.001).

**Table 1:** Demographic and clinical characteristics of risk groups.

	Low-risk category (n = 1,128)	Intermediate-risk category (n = 1,253)	High-risk category (n = 312)	P Value
Age, years	60 (53–68)	62 (53–69)	63 (54–72)	<0.001
Male	883 (78.3%)	941 (75.1%)	217 (69.6%)	0.005
Body mass index, kg/m^2^	24.16 (22.39–26.05)	24.11 (21.97–25.87)	24.02 (22.03–25.69)	0.170
Medical history				
Smoker (former or current)	583 (51.7%)	633 (50.5%)	142 (45.5%)	0.155
Hypertension	525 (46.5%)	602 (48.0%)	151 (48.4%)	0.718
Diabetes mellitus	202 (17.8%)	260 (20.8%)	98 (31.4%)	<0.001
Dyslipidemia	809 (71.7%)	883 (70.5%)	214 (68.6%)	0.532
Previous MI	122 (10.8%)	134 (10.7%)	35 (11.2%)	0.965
Previous PCI	47 (4.2%)	50 (4.0%)	8 (2.6%)	0.422
Previous stroke	46 (4.1%)	65 (5.2%)	22 (7.1%)	0.086
Presentation characteristics				
Heart rate, beats/min	76 (68–78)	76 (69–83)	76 (69–88)	<0.001
SBP, mm Hg	124 (118–135)	123 (111–138)	120 (105–131)	<0.001
Killip class I	960 (85.1%)	970 (77.4%)	227 (72.8%)	<0.001
Killip class II	122 (10.8%)	172 (13.7%)	41 (13.1%)	
Killip class III	42 (3.7%)	93 (7.4%)	27 (8.7%)	
Killip class IV	4 (0.4%)	18 (1.4%)	17 (5.4%)	
Cardiac arrest	6 (0.5%)	16 (1.3%)	7 (2.2%)	0.022
Laboratory findings				
WBC count, ×10^9^/L	5.98 (5.23–6.98)	8.89 (7.21–10.07)	9.10 (8.80–10.77)	<0.001
Neutrophil count, ×10^9^/L	3.79 (3.12–4.53)	6.54 (4.24–8.01)	6.67 (5.64–8.53)	<0.001
Lymphocyte count, ×10^9^/L	1.63 (1.26–2.04)	1.58 (1.18–2.07)	1.63 (1.22–2.16)	0.351
Monocyte count, ×10^9^/L	0.37 (0.28–0.47)	0.49 (0.35–0.71)	0.59 (0.38–0.82)	<0.001
Eosinophil count, ×10^9^/L	0.08 (0.05–0.15)	0.05 (0.01–0.11)	0.04 (0.01–0.10)	<0.001
Basophil count, ×10^9^/L	0.02 (0.01–0.03)	0.02 (0.01–0.02)	0.01 (0.01–0.02)	0.001
RBC count, ×10^12^/L	4.81 (4.53–5.12)	4.69 (4.26–5.14)	4.42 (3.97–4.94)	<0.001
Hemoglobin, g/L	152 (143–161)	148 (131–160)	138 (121–156)	<0.001
RDW, %	13.20 (12.80–13.80)	13.50 (12.90–14.00)	13.70 (13.13–14.40)	<0.001
MCV, fL	92.80 (89.90–96.10)	91.70 (88.60–95.00)	92.10 (88.43–95.60)	<0.001
MCH, pg	31.60 (30.50–32.70)	31.30 (30.10–32.40)	31.20 (29.80–32.20)	<0.001
MCHC, g/L	339 (333–346)	341 (332–349)	337 (328–345)	<0.001
Hematocrit, %	44.80 (42.20–47.40)	43.20 (39.40–46.40)	41.85 (36.53–46.10)	<0.001
Platelet count, ×10^9^/L	168 (131–206)	188 (147–231)	163 (130–210)	<0.001
MPV, fL	11.50 (10.70–12.40)	11.30 (10.50–12.10)	13.30 (12.03–13.80)	<0.001
PDW, fL	14.90 (13.00–16.70)	14.60 (12.80–15.90)	18.30 (14.98–20.40)	<0.001
P-LCR, %	38.20 (32.33–44.40)	37.00 (30.10–42.00)	50.55 (39.39–54.20)	<0.001
Plateletcrit, %	0.210 (0.170–0.230)	0.220 (0.180–0.260)	0.219 (0.180–0.260)	<0.001
LDL, mmol/L	2.40 (1.88–2.98)	2.58 (1.99–3.24)	2.63 (2.09–3.16)	<0.001
Glucose, mmol/L	5.60 (4.89–7.18)	6.20 (5.12–7.98)	6.86 (5.44–9.81)	<0.001
Creatinine, µmol/L	75.75 (67.00–84.00)	74.50 (64.00–87.05)	77.40 (64.00–96.90)	0.071
Elevated cardiac enzymes	521 (46.2%)	898 (71.7%)	236 (75.6%)	<0.001
ST-segment deviation on ECG	450 (39.9%)	810 (64.6%)	209 (67.0%)	<0.001
LVEF, %	56 (55–60)	56 (49–58)	55 (47–59)	<0.001
GRACE risk score	118 (87–166)	162 (114–189)	172 (132–203)	<0.001
ACS presentation				<0.001
NSTE-ACS	724 (64.2%)	513 (40.9%)	125 (40.1%)	
STEMI	404 (35.8%)	740 (59.1%)	187 (59.9%)	
Medical treatment				
Aspirin	1,101 (97.6%)	1,213 (96.8%)	308 (98.7%)	0.136
P2Y12 inhibitor	1,068 (94.7%)	1,197 (95.5%)	299 (95.8%)	0.538
Statin	1,003 (88.9%)	1,135 (90.6%)	275 (88.1%)	0.276
ACEI⁄ARB	761 (67.5%)	796 (63.5%)	201 (64.4%)	0.124
β-Blocker	636 (56.4%)	692 (55.2%)	168 (53.8%)	0.692

Abbreviation: MI, myocardial infarction; PCI, percutaneous coronary intervention; SBP, systolic blood pressure; WBC, white blood cell; RBC, red blood cell; RDW, red blood cell distribution width; MCV, mean corpuscular volume; MCH, mean corpuscular hemoglobin; MCHC, mean corpuscular hemoglobin concentration; MPV, mean platelet volume; PDW, platelet distribution width; P-LCR, platelet large cell ratio; LDL, low-density lipoprotein cholesterol; ECG, electrocardiograms; LVEF, left ventricular ejection fraction; GRACE, Global Registry of Acute Coronary Events; ACS, acute coronary syndrome; NSTE-ACS, non-ST-segment elevation ACS; STEMI, ST-segment elevation MI; ACEI, angiotensin-converting enzyme inhibitor; and ARB, angiotensin-receptor blocker.

Values are expressed as number (percentage) or median (interquartile range).

Low-risk category is defined as patients with WBC count <8.62 × 10^9^/L and a hemoglobin level ≥133 g/L.

Intermediate-risk category is defined as patients with either WBC count (<8.62 × 10^9^/L) + hemoglobin level (<133 g/L) or WBC count (≥8.62 × 10^9^/L) + MPV level (<12.90 fL) + hemoglobin level (≥128 g/L).

High-risk category is defined as patients with either WBC count (≥8.62 × 10^9^/L) + MPV level (≥12.90 fL) or WBC count (≥8.62 × 10^9^/L) + MPV level (<12.90 fL) + hemoglobin level (<128 g/L).

### Relationship between the CART risk categories and clinical outcomes

**Figure 2:**
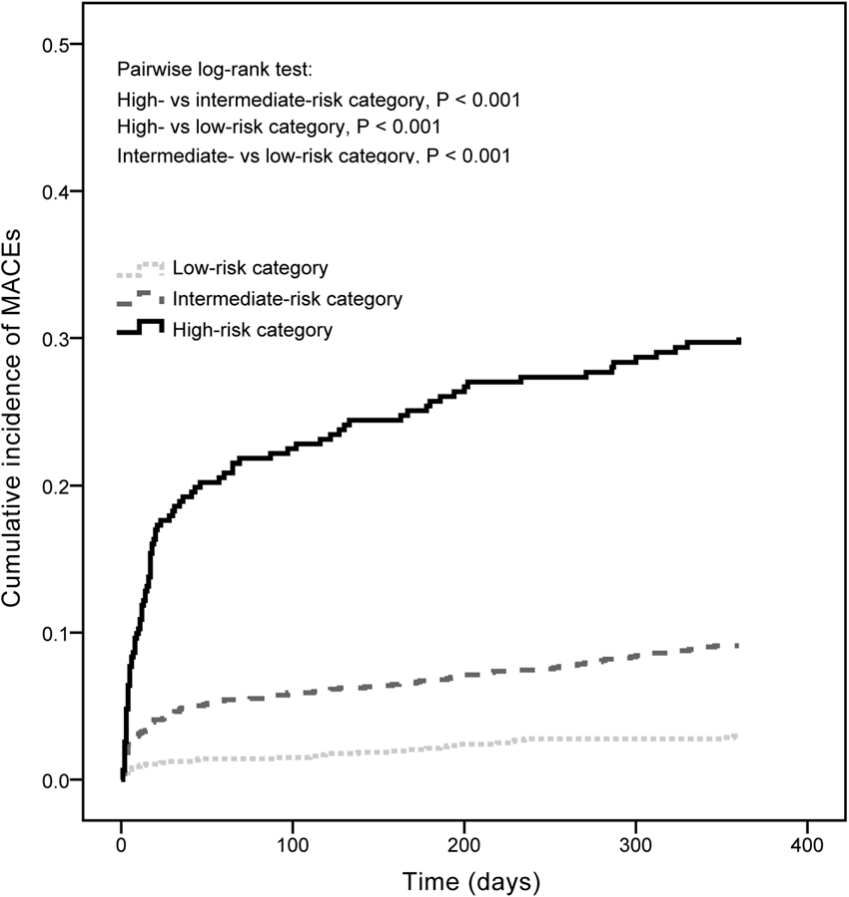
Kaplan-Meier survival analyses of major adverse cardiovascular events (MACEs) at 1-year follow-up by the risk categories. MACE risk increased with the ordering of the risk categories. See Table 1 footnote for the definitions of risk categories determined by the white blood cell count, hemoglobin, and mean platelet volume levels.

The rate of patients lost at the 1-year follow-up was low overall (4.6%) and did not differ by the CART risk categories (low-risk: 4.9%; intermediate-risk: 4.4%; high-risk: 4.2%; P = 0.80). The incidence of MACEs at 1-year follow-up was 3.0, 9.0, and 29.8% for the low-, intermediate- and high-risk categories, respectively (P < 0.001). The Kaplan-Meier curves for the three risk categories demonstrated significantly higher rates of MACEs in the high-risk than in the other two lower risk categories (log-rank test P < 0.001), as shown in Fig. 2.

Results of unadjusted and adjusted Cox models for MACEs are shown in Table 2. In unadjusted analyses, patients in the high-risk and intermediate-risk groups showed increased risks of MACEs when compared to those in the low-risk group (both P < 0.001). An increasing risk stratification from low to high was associated with elevated risk of MACEs (hazard ratio [HR] 3.50, 95% confidence interval [CI] 2.90–4.23). The linear test of trends across the CART risk categories was significant (P_trend_ < 0.001). After adjustment for multiple covariates using five different models, the CART risk categories, which were assessed using tertiles and a linear trend test, remained independent predictors of MACEs in patients with ACS (P < 0.001).

**Table 2:** Unadjusted and adjusted Cox proportional hazards models for major adverse cardiac events according to risk groups and its individual components.

Variables	No adjustment HR (95% CI)	P Value	Model 1^&^HR (95% CI)	P Value	Model 2^¶^ HR (95% CI)	P Value	Model 3^ϕ^ HR (95% CI)	P Value	Model 4^§^ HR (95% CI)	P Value	Model 5^£^ HR (95%CI)	P Value
Risk group analysis^*^												
Low-risk	1.00 (Reference)		1.00 (Reference)		1.00 (Reference)		1.00 (Reference)		1.00 (Reference)		1.00 (Reference)	
Intermediate-risk	3.08 (2.10–4.53)	<0.001	3.00 (2.04–4.40)	<0.001	2.67 (1.53–3.36)	<0.001	2.77 (1.85–4.15)	<0.001	2.18 (1.48–3.20)	<0.001	2.65 (1.76–3.98)	<0.001
High-risk	11.63 (7.85–17.23)	<0.001	10.96 (7.38–16.26)	<0.001	6.26 (4.12–9.53)	<0.001	10.21 (6.32–16.51)	<0.001	6.90 (4.60–10.34)	<0.001	8.15 (5.01–13.24)	<0.001
Risk stratification from low to high	3.50 (2.90–4.23)	<0.001	3.40 (2.81–4.11)	<0.001	2.56 (2.09–3.15)	<0.001	3.27 (2.57–4.17)	<0.001	2.76 (2.26–3.37)	<0.001	2.89 (2.27–3.68)	<0.001
Categorical biomarker analysis												
WBC ≥8.62 × 10^9^/L	3.35 (2.53–4.43)	<0.001	3.36 (2.54–4.44)	<0.001	2.17 (1.61–2.92)	<0.001	3.33 (2.35–4.74)	<0.001	2.17 (1.63–2.89)	<0.001	2.85 (2.00–4.06)	<0.001
Hemoglobin <128 g/L	2.94 (2.20–3.94)	<0.001	2.62 (1.92–3.59)	<0.001	1.77 (1.27–2.48)	0.001	1.69 (1.10–2.59)	0.016	2.23 (1.65–3.00)	<0.001	1.58 (1.02–2.46)	0.042
MPV ≥12.9 fL	2.41 (1.85–3.16)	<0.001	2.45 (1.87–3.20)	<0.001	2.43 (1.85–3.21)	<0.001	3.56 (2.31–5.49)	<0.001	2.53 (1.94–3.31)	<0.001	2.92 (1.88–4.53)	<0.001
Continuous biomarkers analysis												
WBC, per 1 × 10^9^/L increment	1.13 (1.11–1.17)	<0.001	1.14 (1.11–1.17)	<0.001	1.05 (1.01–1.08)	0.011	1.80 (1.54–2.11)	<0.001	1.06 (1.03–1.09)	<0.001	1.45 (1.28–1.65)	<0.001
Hemoglobin, per g/L increment	0.98 (0.97–0.98)	<0.001	0.98 (0.97–0.99)	<0.001	0.99 (0.98–1.00)	0.002	0.97 (0.96–0.99)	0.001	0.98 (0.98–0.99)	<0.001	0.98 (0.96–1.00)	0.020
MPV, per fL increment	1.29 (1.18–1.41)	<0.001	1.30 (1.19–1.42)	<0.001	1.30 (1.19–1.42)	<0.001	1.83 (1.50–2.22)	<0.001	1.30 (1.19–1.42)	<0.001	1.73 (1.41–2.11)	<0.001

^*^See Table 1 footnotes for definitions of risk categories determined by white blood cell (WBC) count, hemoglobin, and mean platelet volume (MPV) levels.

^&^Adjusted for demographic variables (age, gender, and body mass index).

^¶^Adjusted for demographic and clinical covariates (age, gender, body mass index, smoker, diagnosis of hypertension, diabetes, dyslipidemia, history of myocardial infarction, percutaneous coronary intervention, stroke, heart rate, systolic blood pressure, Killip class, cardiac arrest on admission, elevated cardiac enzymes, ST-segment deviation on electrocardiograms, baseline creatinine, glucose, low-density lipoprotein levels, and left ventricle ejection fraction).

^ϕ^Adjusted for other CBC components (neutrophil, lymphocyte, eosinophil, basophil, monocyte, red blood cell count, hematocrit, mean corpuscular volume, red blood cell distribution width, mean corpuscular hemoglobin, mean corpuscular hemoglobin concentration, platelets, platelet distribution width, platelet large cell ratio, and plateletcrit).

^§^Adjusted for the Global Registry of Acute Coronary Events (GRACE) score.

^£^Adjusted for variables that were statistically different among the 3 study groups and established risk factors for adverse outcomes following acute coronary syndrome (age, gender, diabetes, heart rate, systolic blood pressure, Killip class, cardiac arrest on admission, baseline neutrophil, eosinophil, basophil, monocyte, red blood cell count, hematocrit, mean corpuscular volume, red blood cell distribution width, mean corpuscular hemoglobin, mean corpuscular hemoglobin concentration, platelets, platelet distribution width, platelet large cell ratio, plateletcrit, creatinine, glucose, and low-density lipoprotein levels, elevated cardiac enzymes, ST-segment deviation on electrocardiograms, left ventricle ejection fraction, GRACE score, and acute coronary syndrome presentation).

To further validate the predictive ability of the CART algorithm, the association between the three CBC components in the CART analysis and outcomes was determined (Table 2). The three biomarkers (WBC, hemoglobin, and MPV) evaluated as both continuous and categorical variables were significantly associated with higher risk of MACEs in the unadjusted and adjusted Cox models (P < 0.001).

### Discrimination and calibration testing

**Figure 3:**
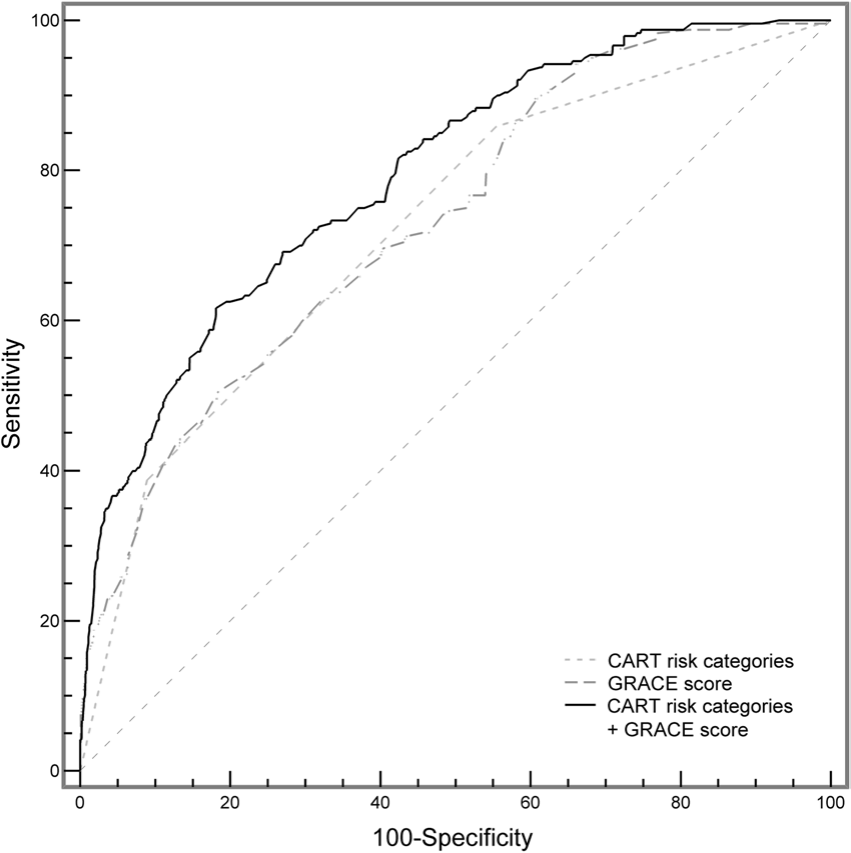
Receiver operating characteristic curve analysis. Addition of the risk categories to the GRACE score improved the predictive value for major adverse cardiovascular events. See Table 1 footnote for the definitions of risk categories determined by the white blood cell count, hemoglobin, and mean platelet volume levels.

**Figure 4:**
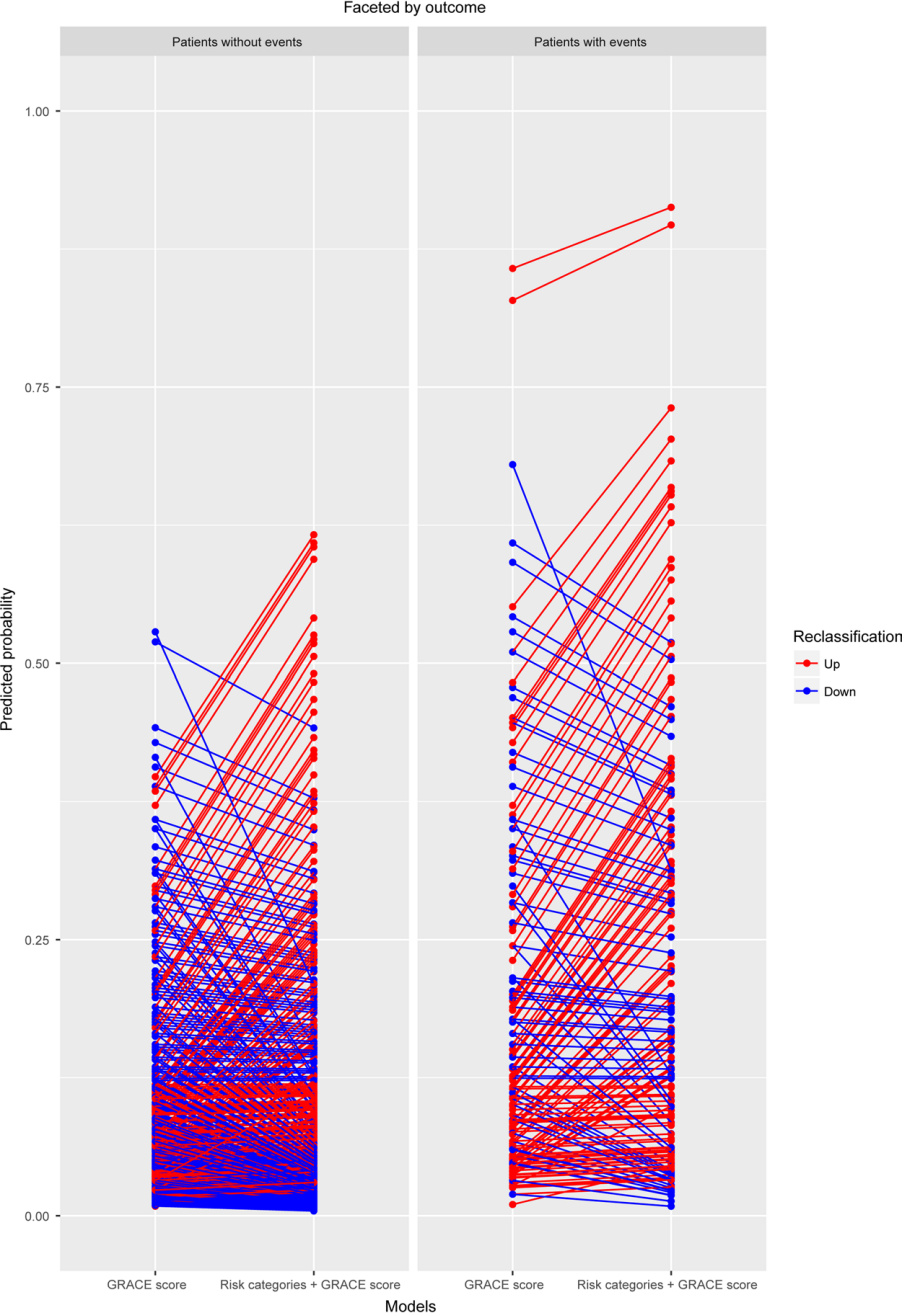
Reclassification by combining the risk categories and GRACE score. The addition of the risk categories to the GRACE score resulted in 38.6% of the patients being correctly reclassified. Decreasing predicted probability is good for patients without adverse events. While increasing predicted probability is good for those with adverse events.

First, we evaluated the usefulness of the CART risk categories in risk discrimination. As shown in Fig. 3, the area under the receiver operating characteristic curve (ROC-AUC) of the CART risk categories for predicting MACEs was 0.721 (95% CI 0.704–0.738). The discriminatory performance of the CART risk categories was similar to the GRACE score (0.732 [95% CI 0.714–0.748]) (P = 0.674). When the CART risk categories were incorporated into the GRACE score, the updated prediction model underwent an improvement in the ROC-AUC to 0.792 (95% CI 0.776–0.807). There was a significant difference in the ROC-AUC between the updated model and GRACE score alone (0.061, 95% CI 0.036–0.086, P < 0.001). With regard to the classification accuracy, by including the CART risk categories into the GRACE score, 38.6% (95% CI 25.7–51.4%) of patients were correctly reclassified (net reclassification improvement [NRI], P < 0.001) (Fig. 4). The integrated discrimination improvement (IDI) was estimated as 0.05 (95% CI 0.04–0.07).

**Figure 5:**
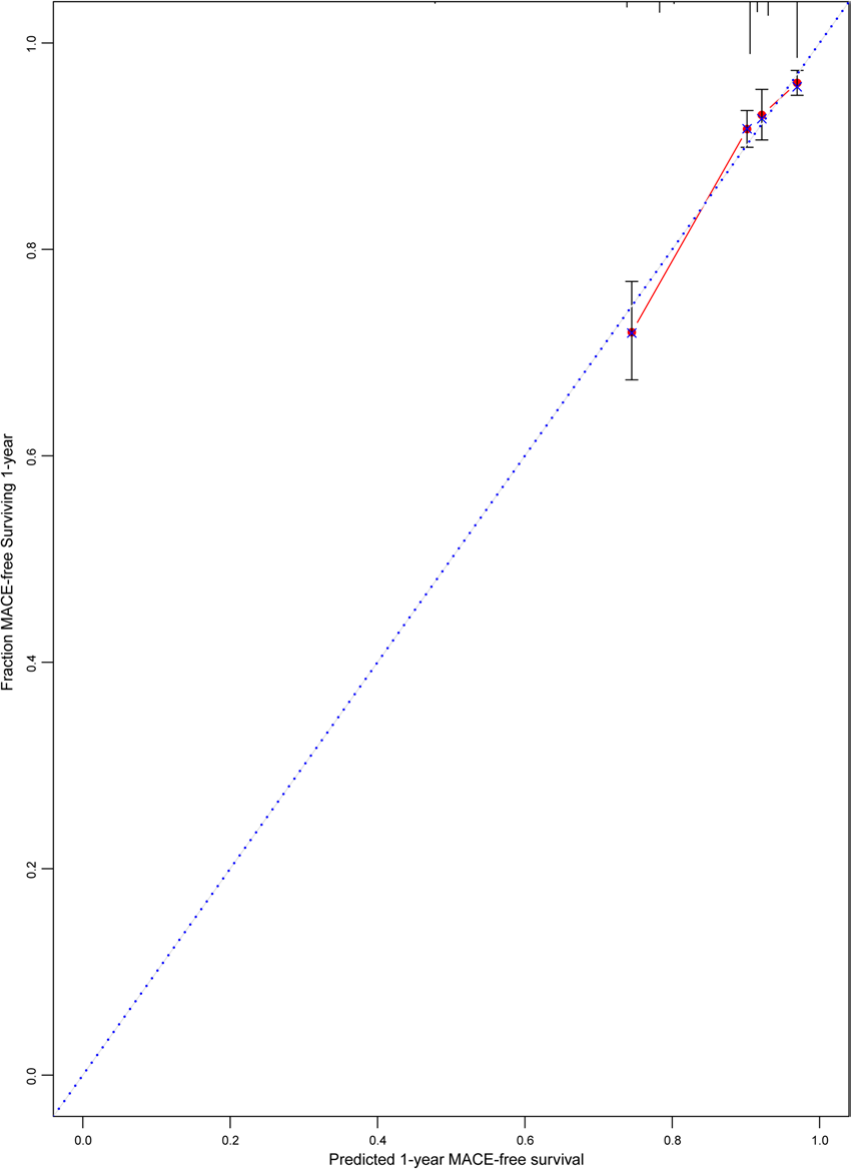
Calibration plot of the risk model. Blue-dashed indicates the optimal calibration line, and red indicates the calibration line obtained from the risk model. See Table 1 footnote for the definitions of risk categories determined by the white blood cell count, hemoglobin, and mean platelet volume levels.

Second, we evaluated the calibration of the CART risk model in patients with ACS. Comparison of the predicted probabilities with the observed frequencies of MACEs at 1-year follow-up demonstrated good calibration, as shown in Fig. 5.

### Subgroup analyses

**Figure 6:**
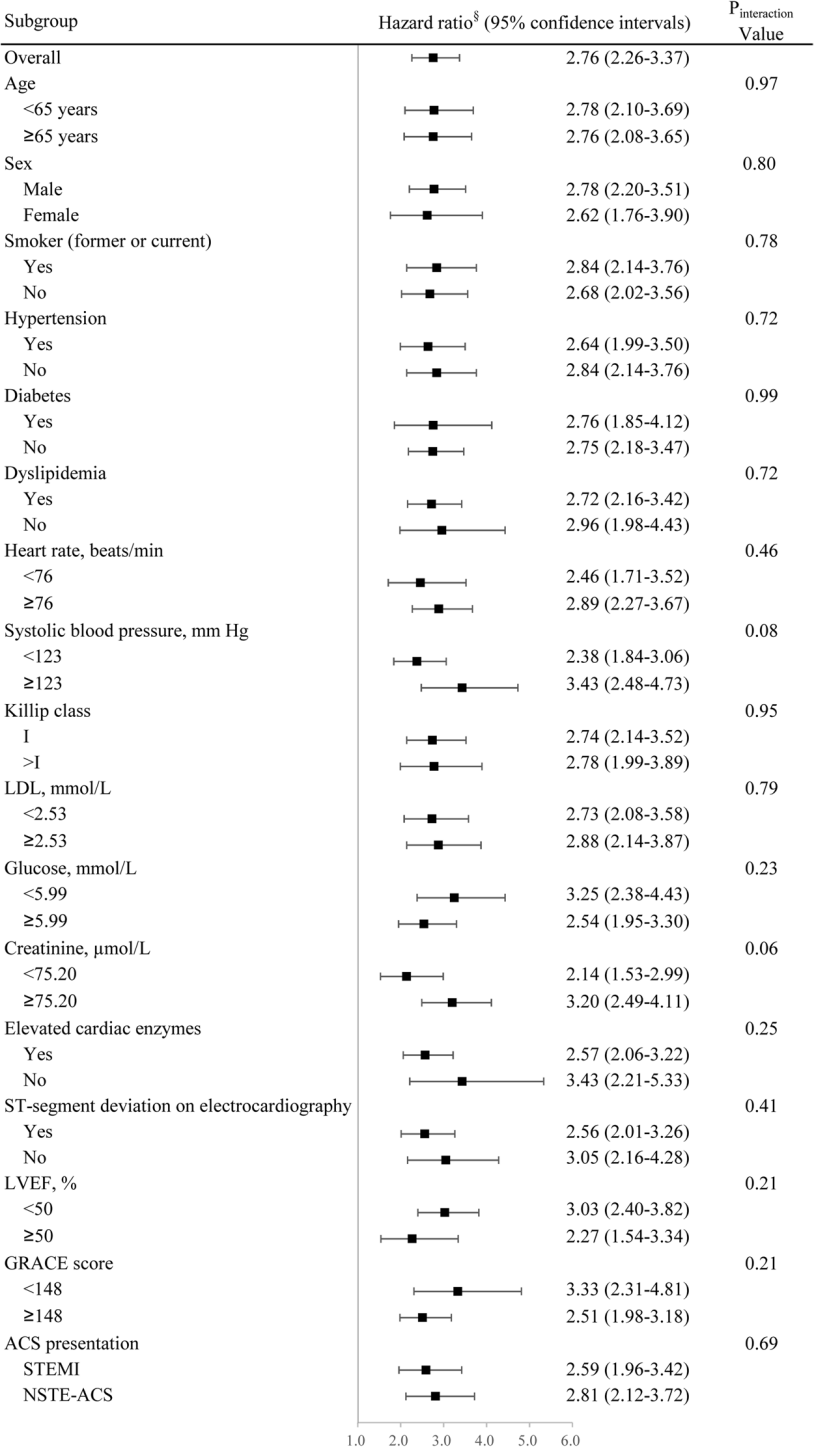
Forest plot of adjusted hazard ratios for the overall and substrata linear trends by the risk categories in the prediction of major adverse cardiac events. LVEF, left ventricular ejection fraction; STEMI, ST-segment elevation myocardial infarction; ACS, acute coronary syndrome; NSTE-ACS, non-ST-segment elevation ACS. See Table 1 footnotes for definitions of risk categories determined by white blood cell count, hemoglobin, and mean platelet volume levels. ^§^Adjusted for the Global Registry of Acute Coronary Events score.

Figure 6 displays a forest plot showing HRs for the linear trend by the CART risk categories after adjusting for the GRACE score in various subgroups. The CART risk categories predicted risk in all relevant subgroups with no evidence for differential associations (P value for interaction >0.05 for all subgroups).

## Discussion

In the present study, the CART method identified three of 18 potential CBC components as the most important prognostic variables. WBC count, hemoglobin, and MPV levels were independent predictors of MACE risk at 1-year follow-up. Patients were stratified into low-, intermediate-, and high-risk categories based on these three variables. The CART risk categories were strongly associated with MACE risk, and had good discrimination and calibration. Moreover, incremental prognostic information was achieved when the CART risk categories were added to the GRACE score.

Clinical risk prediction tools are helpful in informing medical decisions in a diverse patient population^3^. A variety of risk stratification models have been proposed for the prediction of poor outcomes in patients with ACS. Unfortunately, these models often require the collection of extensive data from clinical characteristics and test results, or they have many calculation difficulties for application to the clinical care process^17^. The CBC is the most widely available, relatively inexpensive laboratory test in the early in-hospital period. In certain clinical settings, such as STEMI patients undergoing primary percutaneous coronary intervention (PCI), the CBC may be the only test determined before or during the procedure. The present study developed a simple risk stratification tool on the basis of the CBC measures that can effectively identify patients with ACS at low-, intermediate-, and high-risk for 1-year MACEs. The CART risk stratification model uses existing clinical laboratory data with no additional costs, without the need for either a physician to collect other data in addition to the CBC, or application of a calculator to assist in the risk evaluation of adverse outcomes. Furthermore, the CART risk categories showed similar discrimination capacity as the GRACE score, but with added prognostic value. Therefore, it could be easily and routinely implemented into everyday clinical practice.

Although 18 CBC variables were considered in this study, only three were actually used in construction of the CART. These three indices, namely, the WBC count, hemoglobin, and MPV levels, have previously been demonstrated as independent predictors of adverse outcomes in patients with ACS. The WBC count is an important marker of inflammation measured on routine hemograms, and recent studies demonstrated a persistent association between the WBC count and MACE risk in patients with ACS^9^ or all-comers population undergoing PCI^8^. Several hypotheses have been postulated to explain the mechanism responsible for the association, such as leukocyte-exacerbated coronary thrombus formation, leukocyte-mediated microvascular injury, and release of proinflammatory and vasculotoxic factors^18^. MPV, the most commonly used measure of platelet size, is considered a marker of platelet activation^19^. Increased MPV is associated with high platelet aggregation activity, more thromboxane synthesis and granule secretion, and high expression of adhesion molecules^19^. Studies have reported that elevated MPV levels were closely related to higher incidence of MACEs in patients with ACS^10,20^. Anemia has the potential to worsen the myocardial ischemic insult in ACS, both by decreasing oxygen supply to myocardium downstream of coronary stenosis^21^ and by increasing myocardial oxygen demand through stimulating a greater cardiac output to maintain adequate blood oxygen levels^22^. Anemia may represent larger extent of ischemic myocardium, and was a powerful predictor of ischemic events and cardiovascular mortality in patients with ACS^6^. The excess mortality associated with high hemoglobin concentration has been shown in STEMI patients with high WBC count, whereas the association did exist in those with low WBC count^23^. The interaction between hemoglobin level and WBC count was also observed in our present study. The combined use of the WBC count, hemoglobin, and MPV levels may represent a biochemical-integrated assessment of inflammatory status, thrombotic risk, and the extent of myocardium at risk. The results showing that the WBC count, hemoglobin, and MPV levels were the three predictors providing the best MACE risk discrimination underscored the importance of these pathophysiologic mechanisms in patients with ACS.

Because there are multiple risk factors in the same patient, a meaningful risk factor analysis must consider factors in combination rather than isolation^15^. In a study including 29,526 patients with suspected cardiovascular disease^11^, a CBC score was developed for the prediction of mortality with the use of the multivariable logistic regression coefficients. Results of the prospective cohort study showed the CBC score which concurrently considered the WBC count, platelet count, and four RBC-related metrics (RDW, mean corpuscular volume [MCV], mean corpuscular hemoglobin concentration [MCHC], and hematocrit [HCT]) divided patients into subgroups at markedly different mortality risks (<1% to >14%). The CBC score was significantly superior to models based only on WBC count alone, HCT alone, or traditional risk factors. Furthermore, the CBC score was significantly associated with morbidity endpoints consisted of heart failure, myocardial infarction, coronary artery disease, atrial fibrillation, and other risk factors that may lead to death^12^. These findings suggested that the combined use of CBC parameters may provide excellent risk prediction and clinical acceptance. Previous studies examining the predictive value of the combined use of CBC markers in patients with ACS often interpreted some individual CBC components as a ratio or aggregated a risk score. The elevated WBC count to MPV level ratio was associated with worse outcomes in patients with STEMI^24^ or non-ST-segment elevation ACS (NSTE-ACS)^25^. The role of the aggregate risk score based on the WBC count, hemoglobin, and PDW levels has been assessed in patients with acute MI^26^. This study by Bae *et al*. found that patients with scores of 2 and 3 had increased risk for in-hospital death compared with those with scores of 0 and 1^26^. The aggregate risk score provided an independent prognostic value beyond cardiovascular risk factors, such as age, gender, heart rate, systolic blood pressure, Killip class, creatinine, and myocardial enzyme levels. However, the study did not construct a series of logistic or Cox regression models to consider other CBC markers simultaneously, which may yield more accurate prognostic information^26^. Moreover, the study did not test the model performance (discrimination and calibration), which inform clinicians about the accuracy of the prediction^17^. In the CART analysis, all variables have equal opportunities at each decision level at optimal cut-off values. The CART method can identify importance of variables and search for optimal combinations of variables that best predict outcomes. Results from the CART are presented as tree diagrams similar to the mode of thinking of clinicians in assessing the prognosis. In the present study, we used the CART method to identify the most important predictors from the CBC panel. Three variables, WBC count, hemoglobin, and MPV, were selected to stratify patients into groups at low-, intermediate-, and high-risk for MACEs at 1-year follow-up. To reduce the risk of confounding, we determined the predictive role of the CART risk categories after adjusting for a comprehensive list of characteristics that may bias our results. Both each of the three variables and the CART risk categories independently predicted the risk of MACEs, which indicated the utility of the CART risk categories for identification of high-risk patients. The predictive accuracy of the CART risk categories was validated by measuring discrimination (C statistic of 0.721) and calibration (assessed graphically). Given the wide availability of CBC data, the CART risk model may provide valuable risk information to clinicians.

The GRACE risk score has been demonstrated to a clinically prognostic model in patients with ACS^3,4^. The GRACE score alone had an AUC of 0.732 in our patients, which confirmed the score as a valuable tool for risk assessment. Therefore, the GRACE score was chosen as the reference to test the additive value of the CART risk categories. This added value was evidenced by the significant increase in three complementary statistical metrics (the C-statistic, NRI, and IDI). The GRACE score did not take the CBC components into account as candidate variables during the development of the model^4^. Our study found a significant but weak correlation between the CART risk categories and GRACE score, suggesting that they may reflect some different pathophysiological aspects related to outcomes. It is possible that indicators reflecting other pathophysiological processes of ACS could help to further classify patients into homogeneous subgroups. Therefore, combining the CART risk categories with the GRACE score may be a more valuable model than the GRACE score alone to identify patients with increased risk of adverse outcomes. Consequently, the combined model may help in further optimizing management decisions and improving outcomes in patients with ACS. Additional studies in large-scale populations are required to fully elucidate the clinical utility of combined of the CART risk categories with the GRACE score.

The current study had several limitations. First, this was a retrospective observational analysis. The study findings might have been influenced by some residual confounders, although we attempted to control our results for multiple prognostic factors by means of multivariable and subgroup analyses. Second, the study used only the CBC data on admission. The CBC parameters obtained from a repeated measurement over time may provide additional research ability to stratify risk. Third, given a lower occurrence of MACEs in our entire study population, we did not perform a subgroup analysis using separate end points. Fourth, as the sample size in the study was relatively small, the prognostic value of the CART risk categories needed to be externally validated on large cohorts.

With the help of the optimal combination of WBC count, hemoglobin, and MPV levels as determined by CART analysis, patients with ACS can be quickly and accurately stratified into subgroups with distinct MACE risks at 1-year follow-up. Moreover, the CART risk categories added prognostic information to the GRACE score. Because the CBC is relatively inexpensive and routinely obtained on admission, the CART risk model could be a potentially useful tool for early risk stratification of patients with ACS. Further studies are required to confirm the usefulness of the CART risk categories in clinical practice, such as improvement of the evaluation and, potentially, management and outcomes of patients with ACS.

## Methods

### Patient Populations

From January 2012 to 2016, consecutive patients admitted to the Department of Cardiology of the First Hospital of Lanzhou University with initial diagnoses of ACS were screened. ACS was defined as STEMI and NSTE-ACS, including non-STEMI (NSTEMI) and unstable angina. According to the Universal Definition of Myocardial Infarction^27^, STEMI was diagnosed in patients showing a rise and/or fall of cardiac biomarker values with at least one value above the 99^th^ percentile of the upper reference limit and at least one of the following: ischemic symptoms, new-onset ST-segment elevation or left bundle-branch block in the index or subsequent ECG, pathological Q waves on ECG, and/or imaging evidence indicative of new ischemia. Cases of NSTEMI required at least one instance of positive cardiac biochemical marker of necrosis without new ST-segment elevation observed on ECG^28^. Unstable angina was defined as rest, new-onset, or worsening angina, respectively, with or without ischemic changes on the ECG and normal myocardial enzymes^28^. The exclusion criteria included (1) cancer or hematological proliferative diseases, (2) autoimmune disease, (3) systemic infection, (4) end-stage liver or renal failure, and (5) no CBC measurements available. The 2,693 patients who met inclusion criteria were enrolled in the analysis. The study was performed in accordance with the Declaration of Helsinki and approved by institutional review board of the First Affiliated Hospital of Lanzhou University with informed consent obtained.

### CBC Measurements and the GRACE Risk Score

Peripheral venous blood samples were drawn upon admission to the hospital. Blood samples were collected in tubes containing dipotassium ethylenediaminetetraacetic acid and were assessed using a Sysmex XE-2100 hematology analyzer (Sysmex, Kobe, Japan). CBC metrics included leukocyte counts (total WBC count and its subtypes [neutrophil, lymphocyte, eosinophil, basophil, and monocyte]), erythrocyte measures (red blood cell [RBC] count, HCT, MCV, RDW, hemoglobin, MCHC, mean corpuscular hemoglobin [MCH], platelets, MPV), and thrombocyte assessments (platelets, MPV, platelet distribution width [PDW], platelet large cell ratio [P-LCR], and plateletcrit).

The GRACE risk score was derived from eight variables that were available on admission (age, heart rate, systolic blood pressure, serum creatinine concentration, Killip class, cardiac arrest, ST-segment deviation, and elevated cardiac enzymes)^4^. Values for these variables were entered into a GRACE risk calculator (www.outcomes-umassmed.org/grace/) to obtain scores for each patient. The GRACE score was originally designed to estimate the cumulative risks of all-cause mortality or nonfatal MI in the period from admission to six months, and it has been shown to have good predictive value for cardiovascular events within 1-year of admission^5,29^.

### Clinical Outcomes

The outcome of interest was MACEs defined as a composite of all-cause mortality, nonfatal MI, stroke, heart failure, or ischemia-driven revascularization at 1-year follow-up. Investigators who were unaware of the aims of the study and blinded to laboratory findings performed the follow-up via medical records or telephone contact.

### Data Collection

Trained investigators collected data by reviewing hospital medical records using a standardized case report form. Data extraction included information regarding demographic characteristics, medical histories, presentation features, laboratory and echocardiographic parameters, medication, and clinical outcomes.

### CART Analysis

The CART analysis was used to identify the best predictors of adverse outcomes and develop the risk stratification model. The CART analysis can establish a binary-branching tree to classify patients into homogeneous subgroups by using recursive partitioning and regression techniques^30^. The CART method involves two steps: First, the tree branches are divided into two child nodes that contain a subgroup of samples from a root node that includes all samples. The child nodes in turn can become parent nodes producing additional child nodes until some minimum subgroup size is reached. The criterion for branching is selected based on the Gini Index after examining every value of each candidate variable. Second, the tree structure is pruned from the bottom of the tree until the tree fits without overfitting the information contained in the data set. The CART method is a nonparametric procedure that can handle continuous variables that are highly skewed and categorical data with either a nominal or ordinal structure. For our study, the independent variable included 18 CBC components, namely total WBC count and its subtypes (neutrophil, lymphocyte, eosinophil, basophil, and monocyte), RBC count, HCT, MCV, RDW, hemoglobin, MCH, MCHC, platelets, MPV, PDW, P-LCR, and plateletcrit. The minimum size of cases in parent node and final child nodes were 100 and 50, respectively. A 10-fold cross-validation, where each run consisted of 10 random partitions of the samples into 90% training and 10% test sets, was used to prune the tree. The MACE rate was calculated for each of the terminal nodes in the CART analysis. Branch points of the CART dividing patients into categories with a higher incidence of MACE were considered as positive biomarkers. Patients were further classified into low-, intermediate-, and high-risk categories according to the number of positive biomarkers. The predictive value of the risk stratification model was determined using univariable and multivariable Cox proportional hazards models. The model discrimination was assessed by calculating the ROC-AUC (C-statistic). The ability of the CART risk stratification to more accurately stratify individuals into higher or lower risk categories (reclassification) was evaluated by using the NRI and IDI metrics. The calibration of the risk categories were tested by comparing the predicted with the observed outcomes in each quartile of predicted risk by CBC variables in the CART analysis.

### Statistical Analyses

The Kolmogorov-Smirnov test was used to assess the normal distribution of quantitative variables. Continuous variables are reported as medians with interquartile ranges and compared with Kruskal-Wallis test, as all data were non-normally distributed. Categorical variables are presented as frequencies and percentages and compared with χ^2^ test, Fisher exact test, or linear-by-linear association, as appropriate. The relationship between the CART risk categories and the GRACE score was assessed by Spearman rank correlation. The Kaplan-Meier method was used to analyze the cumulative incidence of events among the different risk categories during follow-up, and the pairwise log-rank test was performed to assess the difference between the groups. The relationship between the CART risk categories and outcomes was determined using Cox regression in unadjusted models and in five different models adjusted for demographic variables (model one: age, gender, and body mass index), demographic variables plus clinically relevant covariates for MACE outcomes (model two: age, gender, body mass index, smoker, diagnosis of hypertension, diabetes, dyslipidemia, history of MI, PCI, stroke, heart rate, systolic blood pressure, Killip class, cardiac arrest on admission, elevated cardiac enzymes, ST-segment deviation on ECG, baseline creatinine, glucose, low-density lipoprotein levels, and left ventricle ejection fraction [LVEF]), other CBC components (model three: neutrophil, lymphocyte, eosinophil, basophil, monocyte, RBC count, HCT, MCV, RDW, MCH, MCHC, platelets, PDW, P-LCR, and plateletcrit), the GRACE risk score (model four), or the variables that were statistically different among the 3 risk groups plus other variables known to affect prognosis after ACS (model five: age, gender, diabetes, heart rate, systolic blood pressure, Killip class, cardiac arrest on admission, baseline neutrophil, eosinophil, basophil, monocyte, RBC count, HCT, MCV, RDW, MCH, MCHC, platelets, PDW, P-LCR, plateletcrit, creatinine, glucose, and low-density lipoprotein levels, elevated cardiac enzymes, ST-segment deviation on ECG, LVEF, GRACE score, and ACS presentation). In the Cox regression analysis, the CART risk categories were modeled as either nominal (individual groups) or ordinal (a linear trend test) categorical variables. Additionally, individual CBC biomarkers in the CART risk categories were also evaluated both as continuous and as categorical variables based on cut-off points identified with the CART method. Results are reported as HRs with 95% CIs. The predictive accuracy of the CART risk categories alone, and the combined CART risk categories and the GRACE score, were estimated by applying DeLong’s test to compare the AUC from each of the models^31^. The continuous NRI and IDI measures were also computed to assess the increased discriminative power after the addition of the CART risk categories to the GRACE score by using the PredictABEL package in R^32^. The continuous NRI determines net percentages of patients who do and do not have events who were correctly reclassified using the updated model^33^. In graphical representation of the continuous NRI, for those without events, decreasing predicted risk is good; for those with events, increasing predicted risk is good. The IDI is equal to the difference of initial and updated models in discrimination slope formed between the mean predicted probabilities in those with and without events^33^. Calibration plot was made using the rms package in R^34^. Subgroup analyses were conducted to examine whether the prognostic value of the CART risk categories varied according to specified factors including age (<65 years or ≥65 years), sex, smoker, diagnosis of hypertension, diabetes, dyslipidemia, heart rate (<median or ≥median), systolic blood pressure (<median or ≥median), Killip class (I or >I), low-density lipoprotein level (<median or ≥median), glucose level (<median or ≥median), creatinine level (<median or ≥median), elevated cardiac enzymes, ST-segment deviation on ECG, LVEF (<50% or ≥50%), GRACE score (<median or ≥median), and ACS presentation (STEMI or NSTE-ACS). We evaluated effect modification using interaction terms between subgroups. A 2-tailed P < 0.05 was considered significant. All analyses were performed with SPSS version 22.0 (SPSS, Chicago, Illinois), MedCalc version 8.0.1.0 (MedCalc Software, Mariakerke, Belgium), and R version 3.3.1 (R Foundation for Statistical Computing, Vienna, Austria).

### Data availability statement

All data generated or analysed during this study are included in this published article.
